# A novel fragment derived from the *β* chain of human fibrinogen, *β*43–63, is a potent inhibitor of activated endothelial cells *in vitro* and *in vivo*

**DOI:** 10.1038/sj.bjc.6605495

**Published:** 2010-01-12

**Authors:** E Krajewska, C E Lewis, Y-Y Chen, A Welford, S Tazzyman, C A Staton

**Affiliations:** 1 Tumor Targeting Group, University of Sheffield Medical School, Sheffield S10 2RX, UK; 2 Tumour Microcirculation Group, University of Sheffield Medical School, Sheffield S10 2RX, UK; 3 Microcirculation Research Group, University of Sheffield Medical School, Sheffield S10 2RX, UK

**Keywords:** fibrinogen, *β*43–63, endothelial, anti-angiogenic, haemostasis

## Abstract

**Background:**

Angiogenesis and haemostasis are closely linked within tumours with many haemostatic proteins regulating tumour angiogenesis. Indeed we previously identified a fragment of human fibrinogen, fibrinogen E-fragment (FgnE) with potent anti-angiogenic properties *in vitro* and cytotoxic effects on tumour vessels *in vivo*. We therefore investigated which region of FgnE was mediating vessel cytotoxicity.

**Methods:**

Human dermal microvascular endothelial cells (ECs) were used to test the efficacy of peptides derived from FgnE on proliferation, migration, differentiation, apoptosis and adhesion before testing the efficacy of an active peptide on tumour vasculature *in vivo*.

**Results:**

We identified a 20-amino-acid peptide derived from the *β* chain of FgnE, *β*43–63, which had no effect on EC proliferation or migration but markedly inhibited the ability of activated ECs to form tubules or to adhere to various constituents of the extracellular matrix – collagen IV, fibronectin and vitronectin. Furthermore, our data show that *β*43–63 interacts with ECs, in part, by binding to *α*_v_*β*_3_, so soluble *α*_v_*β*_3_ abrogated *β*43–63 inhibition of tubule formation by activated ECs. Finally, when injected into mice bearing tumour xenografts, *β*43–63 inhibited tumour vascularisation and induced formation of significant tumour necrosis.

**Conclusions:**

Taken together, these data suggest that *β*43–63 is a novel anti-tumour peptide whose anti-angiogenic effects are mediated by *α*_v_*β*_3_.

Angiogenesis, the outgrowth of new capillaries from the existing vasculature, is a multistep process that involves cell proliferation, migration and differentiation (Folkman, 1995). It is a critical step in tumour growth and is tightly regulated by a change in balance between pro-angiogenic and anti-angiogenic factors in the local environment. This often involves an upregulation in the expression of pro-angiogenic factors, such as vascular endothelial growth factor (VEGF), epidermal growth factor (EGF), platelet-derived growth factor (PDGF) and type II fibroblast growth factor (FGF2) (Folkman, 1995).

Many peptides derived from proteins involved in haemostasis have been shown to regulate tumour angiogenesis (Daly *et al*, 2003). The blood clotting factor, fibrinogen (Fgn), is a large (340 kDa) protein that contains three pairs of nonidentical peptides: the *α*-, *β*- and *γ* chains. These are arranged into three domains, the two outer D-domains and a central E-domain (Doolittle, 1981). Fibrinogen often accumulates in and around leaky blood vessels in tumours and promotes tumour angiogenesis by supporting cell adhesion, migration, proliferation and differentiation of activated endothelial cells (ECs) (Zacharski *et al*, 1986). Fibrinogen can be digested in the body by plasmin and when this occurs each Fgn molecule gives rise to two D fragments, a number of small fragments, including a small peptide, *β*1–42 (the N terminus of the *β*-chain) and one 50-kDa E-fragment (called fibrinogen E-fragment; FgnE) consisting of the N-terminal regions of the *α*, *β* and *γ* chains held together by disulphide bonds (Bootle-Wilbraham *et al*, 2000).

We previously reported that FgnE is a potent anti-angiogenic factor *in vitro* and vascular-damaging agent *in vivo*. It inhibits the migration and differentiation of human ECs in response to VEGF, EGF and FGF2 *in vitro* (Bootle-Wilbraham *et al*, 2000) and is cytotoxic for activated ECs *in vitro*. These effects may explain why FgnE selectively disrupts tumour endothelium, causing widespread intravascular thrombosis and tumour necrosis *in vivo* (Brown *et al*, 2002). We also showed that alphastatin, a 24-amino-acid peptide fragment derived from the N terminus of the *α* chain of FgnE, mimics many of the anti-angiogenic effects of FgnE *in vitro* as well as its anti-vascular effects *in vivo*. However, it is not cytotoxic for activated EC unlike FgnE (Staton *et al*, 2004).

We show here that another peptide found in the N terminus of the *β* chain of FgnE (amino acids 43–63, Figure 1A) also has minimal effects on EC viability but displays a pronounced ability to inhibit the adherence of activated ECs to various components of the extracellular matrix (ECM). Furthermore, we show that it does so by binding to, and blocking ECM binding of, the integrin *α*_v_*β*_3_ on ECs. Moreover, when injected into tumour-bearing mice, this peptide inhibited tumour vascularisation and caused increased levels of tumour necrosis.

## Materials and methods

### Cells

Adult human dermal microvascular endothelial cells (HuDMECs) were obtained commercially (PromoCell, Heidelberg, Germany) and cultured in microvascular endothelial cell growth medium (EGM) with supplements (consisting of 0.4% CGS/H, 5% fetal calf serum (FCS), 10 ng ml^−1^ EGF, 1 *μ*g ml^−1^ hydrocortisone, 50 ng ml^−1^ amphotericin B, 50 *μ*g ml^−1^ gentamicin). Cells were grown at 37°C in a 100% humidified incubator with the gas phase of 5% CO_2_ and routinely screened for *Mycoplasma*. Human dermal microvascular endothelial cells were used in the experiments until they reached passage 6.

### Proteins and peptides

All chemicals and reagents used were obtained from Sigma-Aldrich (Dorset, UK) unless stated otherwise, and were of AnalaR or high-performance liquid chromatography grade. Recombinant human growth factors (VEGF165, EGF, PDGF, HGF and FGF2) were purchased from R&D systems (Abingdon, UK), commercial human FgnE and recombinant human *α*_v_*β*_3_ were purchased from Calbiochem (Merck, Darmstadt, Germany). *β*43–63 (ARPAKAAATQKKVERKAPDA), the control peptide, a scrambled form of *β*43–63 (scrambled control peptide (*β*SC): RAQVPPAKKDAARATKAKAE) and other peptides derived from FgnE, including alphastatin, were synthesised by GenScript Corporation (Piscataway, NJ, USA). In all experiments, FgnE was used at a final concentration of 1 *μ*M and the peptides used at 2 *μ*M (which corresponds to 1 *μ*M FgnE).

### Tubule formation assay

A total of 96-well plates were coated with 34 *μ*l per well of growth factor-reduced (GF-reduced) Matrigel (BD Biosciences, Bedford, MA, USA). Human dermal microvascular endothelial cells were serum depleted overnight in EGM+1%FCS and then seeded at 1.5 × 10^4^ cells per well and allowed to attach for 45 min. The medium was then replaced with treatment medium (EGM+1%FCS), with/without peptides and/or GFs: VEGF (10 ng ml^−1^), EGF (35 ng ml^−1^), FGF2 (25 ng ml^−1^), PDGF-BB (25 ng ml^−1^) and hepatocyte growth factor (HGF; 100 ng ml^−1^). Endothelial cells on this matrix migrated and formed tubules within 6 h of plating (Kleinman *et al*, 1986). Tubule formation was monitored at × 40 magnification, using a light microscope (Nikon Eclipse TS100, KingsTon upon Thames, Surrey, UK) and analysed using the image analysis package, Scion Image (Frederick, MA, USA) as previously described (Bootle-Wilbraham *et al*, 2000). For the experiments using *α*_v_*β*_3_, the *β*43–63 was incubated with recombinant *α*_v_*β*_3_ for 1 h before addition to the Matrigel assay.

### Migration assay

The migration assay, adapted from Malinda *et al* (1999) involved the use of a 48-well microchemotaxis chamber (Neuro Probe, Cabin John, MD, USA) with 8 *μ*m pore size polycarbonate membranes (Neuro Probe) coated with 100 *μ*g ml^−1^ human collagen type I or IV. Vascular endothelial growth factor alone (10 ng ml^−1^) or with peptides/FgnE was dissolved in EGM+1% FCS and placed in the bottom wells. The membrane was then positioned and 50 *μ*l of 2.5 × 10^5^ HuDMECs per ml (serum depleted in EGM+1%FCS overnight) was added to the top chamber. The chambers were incubated at 37°C for 4.5 h then migrated cells on the bottom surface were fixed and stained with Hema ‘Gurr’ rapid staining kit (VWR, Poole, UK) and counted at × 40 magnification in three random fields per well in three replicate wells and repeated three times.

### Proliferation assay

To assess proliferation of HuDMECs, we used the BrdU assay (Merck, Calbiochem, Beeston, UK), whereby BrdU is incorporated into newly synthesised DNA strands of newly proliferating cells. Human dermal microvascular endothelial cells were seeded into 96-well plates at 5 × 10^3^ cells per well in EGM with 1% fetal bovine serum and then incubated with FgnE and peptides for 24 h as above. BrdU label was added for 6 h and then cells were permeabilised and incubated with antibodies according to manufacturers’ instructions. The absorbance was read at 450 nm, using an Anthos Labtec Instruments (Salzburg, Austria) plate reader.

### Cell viability assays

Human dermal microvascular endothelial cells were seeded into a 48-well plate at a density of 2 × 10^5^ cells per well and then serum starved for 24 h in EGM+1%FCS. The cells were then incubated for 24 h with *β*43–63 or *β*SC before a 20-min incubation with 1 *μ*l of fluorescein isothiocyanate (FITC)-conjugated Z-VAD-FMK (carbobenzoxy-valyl-alanyl-aspartyl-fluoromethylketone; Promega, Southampton, UK), a cell permeable, irreversible pan-caspase inhibitor that allows *in situ* labelling of activated caspases. The cells were then washed 3 × with FACS buffer (phosphate-buffered saline (PBS) with 0.1% BSA), trypsynised and finally 1 *μ*l propidium iodide (PI, 50 *μ*g ml^−1^) was added to measure dead cells. A total of 10 000 cells were analysed using FACScan (FL1 and FL2 filters – 530 and 585 nm). The distribution of live, apoptotic and late apoptotic/necrotic cells were analysed based on distribution of staurosporine-treated (400 nM, 2 h) cells that were used as the positive control for apoptosis/necrosis. Live cells had no stain at all, early apoptosis cells were stained with FITC stain only, late-stage apoptotic cells with FITC and PI.

### Western blotting for phosphorylated Akt

Human dermal microvascular endothelial cells were plated down into six-well plates and grown to near confluence before serum depletion (EGM+1%FCS) overnight. The cells were then incubated with peptides for 3 h before a 5-min incubation with VEGF (10 ng ml^−1^). After treatment the cells were washed twice in PBS and suspended in a triple cell lysis buffer (50 nM Tris-HCI (pH 8.5), 150 nM NaCl, 0.1% SDS, 1% nonident-P40, 0.5% sodium deoxycholate and a complete protease inhibitor tablet (Roche, Mannheim, Germany)) and placed on ice for 20 min before centrifugation. Protein concentrations of the supernatants were determined using the bicinchoninic acid assay. Equal amounts of protein extracts (20 *μ*g) were separated by SDS–polyacrylamide gel electrophoresis, electrotransferred to nitrocellulose membranes and exposed to anti-Akt or phospho-Akt (BD Biosciences). Immunoreactive bands were detected by enhanced chemiluminescence (Amersham, Little Chalfont, Buckinghamshire, UK), and band intensity quantified using densitometry software (Bio-Rad, Hemel Hempstead, Hertfordshire, UK).

### Cell adhesion assay

A total of 96-well plates were coated overnight with 10 *μ*g ml^−1^ human collagen type IV (Sigma-Aldrich), 5 *μ*g ml^−1^ human fibronectin (Merck) or 0.5 *μ*g ml^−1^ human vitronectin (Invitrogen, Paisley, Renfrewshire, UK), then washed with PBS. Meanwhile, HuDMECs were serum depleted in EGM+1%FCS overnight then lifted using non-enzymatic cell dissociation solution, and incubated for an hour at 37°C with VEGF (10 ng ml^−1^) in the absence or presence of FgnE, alphastatin, *β*43–63 or *β*SC. Matrix-coated 96-well plates were seeded with 100 *μ*l of cell suspension (5000 cells per well) together with noncoated plates (referred to as ‘plastic control’). After 1 h incubation at 37°C the wells were washed twice with PBS, and any cells that remained attached were fixed with methanol and stained with methyl blue. Stained cells were photographed (at × 40 magnification) and counted in three different fields of view per well.

As integrins mediate cell adhesion to ECM proteins – and the only integrin known to mediate adhesion to all three ECM proteins studied is *α*_v_*β*_3_ – the role of this integrin in mediating the inhibiting effect of *β*43–63 on ECs was investigated. Moreover, the *β* chain of Fgn is thought to bind to heparin sulphate proteoglycans so the effect of heparin on cell adhesion was also investigated. 96-well plates were coated with 100 *μ*l of *β*43–63 or *β*SC overnight (10 *μ*g ml^−1^), then washed and pre-incubated for 1 h with or without recombinant *α*_v_*β*_3_ or heparin before addition of the HuDMECs (pre-incubated with VEGF). The cells were allowed to adhere for 1 h before washing and staining as before.

### Direct binding assays

96-well plates were coated overnight with PBS (plastic control) 10 *μ*g ml^−1^ *β*43–63, 10 *μ*g/ml *β*SC (negative control) or 0.5 *μ*g ml^−1^ vitronectin (positive control), then washed and blocked with 5% BSA in PBS for 1 h. The wells were then washed, incubated with recombinant *α*_v_*β*_3_ for 1.5 h, then washed and incubated with anti-*α*_v_*β*_3_ antibody for 1.5 h, then washed and incubated with anti-mouse-HRP-conjugated secondary antibody for a further hour and finally washed and incubated with OPD for 15 min before reading on an ELISA plate reader (450 nm). Controls included wells coated and treated with everything except *α*_v_*β*_3_ (data shown) and nonspecific binding to plastic was subtracted from the final results.

### Tumour xenograft studies

All experiments were performed under HO Project Licence Number PPL40/3110 and conformed to the United Kingdom Co-ordinating Committee on Cancer Research Guidelines for the Welfare of Animals in Experimental Neoplasia. The CaNT murine mammary adenocarcinoma is a transplanted tumour model, derived from a spontaneous tumour and is maintained *in vivo* by the transfer of tumour cells between mice (Collingridge and Chaplin, 2001). CBA/Gy mice (Gray cancer Institute, Northwood, UK) were anaesthetised by isofluorane inhalation and inoculated subcutaneously with 10^6^ viable CaNT tumour cells. Tumour volume was determined at regular intervals by caliper measurement (accurate to 0.1 mm) in two dimensions as described previously (Brown *et al*, 2002) using the equation: volume=(*a*^2^ × *b*)/2, where *a* is the smaller and *b* the larger diameter of the two. When tumours had grown to 100–150 mm^3^, mice were injected i.p. daily for 10 days with *β*43–63 (0.084 mg kg^−1^) or vehicle (PBS).

After 10 days, mice were killed and tumours (and various normal tissues) excised, divided into two halves and fixed in either (1) 10% neutral buffered formalin or (2) zinc-based fixative (Beckstead, 1994), then processed into paraffin wax. Formalin-fixed sections were stained for H&E and tumour necrosis assessed semi-quantitatively using a Chalkley grid method (% necrosis) (Brown *et al*, 2002). Zinc-fixed sections were exposed to a rat monoclonal anti-murine CD31 (1 : 100; Pharmingen, San Diego, CA, USA) specific for ECs, for 60 min at room temperature, and immunoreactivity detected using the ABC rat elite kit (Vector Laboratories, UK) and diaminobenzidine. Vessels were then counted per field of view ( × 40) for all tumours.

### Statistical analysis

All experiments were performed at least three times, and data were analysed using the Mann–Whitney *U*-test, a nonparametric test that does not assume a Gaussian distribution in the data being analysed. *P*⩽0.05 was taken as significant.

## Results

**Figure 1:**
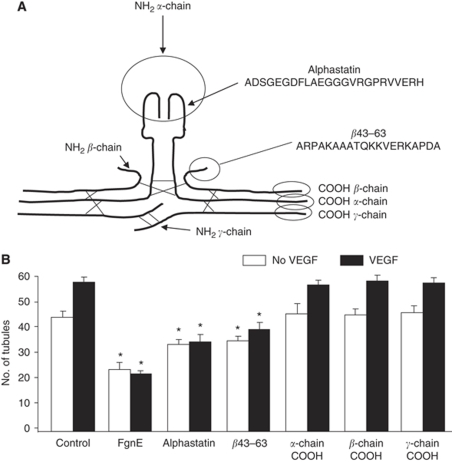
(**A**) Schematic illustration of FgnE. Fibrinogen consists of two *α* chains, two *β* chains and two *γ* chains. Alphastatin is the first 24 amino acids of the N terminus of the *α* chains (*α*1–24) whereas *β*43–63 is the first 20 amino acids on the N terminus of the two *β* chains. 20-amino-acid peptides were also generated from the C terminus of each chain representing the regions exposed upon plasmin cleavage of the original fibrinogen molecule. (**B**) Effects of FgnE and derived peptides on the Matrigel assay. FgnE, alphastatin and *β*43–63 inhibit tubule formation in response to VEGF stimulation as measured by number of tubules per field of view. FgnE was used at 1 *μ*M, but the peptides were used at 2 *μ*M as two copies of these peptides are present in a single FgnE molecule. Data are presented as mean±s.e.m. ^*^P<0.05 with respect to relevant control.

Our previous experiments showed that 1 *μ*M FgnE inhibited differentiation of ECs into tubule-like structures when plated on Matrigel in the presence or absence of VEGF. In contrast the parent Fgn molecule did not cause inhibition (Bootle-Wilbraham *et al*, 2000). We therefore initially synthesised peptides derived from the termini of the FgnE chains exposed by plasmin cleavage (Figure 1A) for testing in the Matrigel assay (Figure 1B). Of the four peptides tested, only *β*43–63 inhibited EC differentiation and was therefore subjected to more thorough investigation.

**Figure 2:**
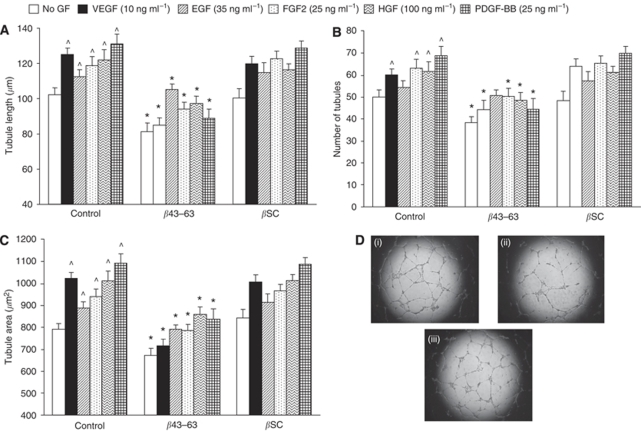
*β*43–63 also inhibits tubule formation by ECs in response to four other pro-angiogenic growth factors, PDGF, FGF2, EGF and HGF, *in vitro*. (**A**) Length, (**B**) number and (**C**) area covered by tubules formed by HuDMECs in response to the above growth factors in the absence or presence of *β*43–63 or scrambled *β*43–63 (*β*SC) used at 2 *μ*M. ^*^P<0.05 with respect to relevant control group, ^*P*<0.05 with respect to control No-GF group. All data are means±s.e.m. Data are representative of three replicate experiments. (**D**) Typical appearance of VEGF-induced tubules (in wells of a 96-well plate) in the presence of (i) VEGF alone, (ii) VEGF + 2 *μ*M *β*43–63 and (iii) VEGF + 2 *μ*M *β*SC.

Indeed Figure 2 shows that *β*43–63, but not the control peptide (scrambled *β*43–63), significantly (*P*<0.05) inhibited the length, number and area of tubules formed by EC activated by the five prominent angiogenic factors found in tumours (VEGF, PDGF-BB, EGF, FGF2 and HGF) on GF-reduced Matrigel, although *β*43–63 showed only minimal inhibition of EGF-induced tubule formation. The inhibitory effects of *β*43–63 were seen for both GF and non-GF activated cells. At first this may appear to suggest that ECs do not need to be ‘activated’ by a stimulant like VEGF to respond to these two agents. However, GF-reduced Matrigel does itself act as a stimulant/activator inducing ECs to form tubules in the absence of exogenous GFs.

**Figure 3:**
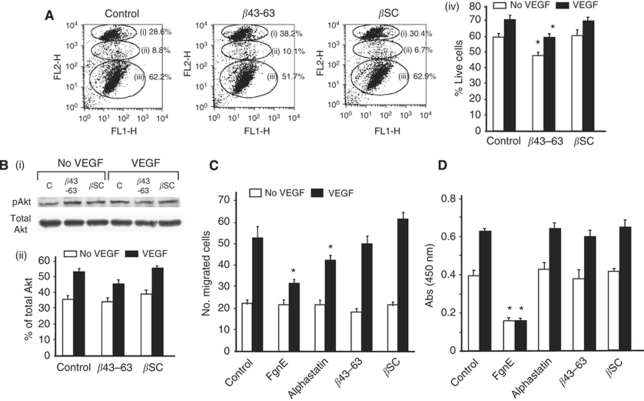
Effects of *β*43–63 on (**A**) viability, (**B**) Akt phosphorylation, (**C**) migration and (**D**) proliferation of HuDMECs in response to VEGF *in vitro*. All data are means±s.e.m. (**A**) Flow cytometry profiles showing (i) late stage apoptosis and necrosis (ii), apoptosis (iii) and live cells in control media or in the presence of *β*43–63 or *β*SC. (iv) graph showing % live cells in presence or absence of VEGF in response to peptide treatment. (**B**) Western blot analysis of Akt phosphorylation in the presence or absence of VEGF (10 ng ml^−1^) in response to peptide treatment. (i) Scans of original blots for phosphorylated Akt and total Akt. (ii) Densitometry analysis showing slight inhibition in phosphorylation of Akt in the presence of VEGF (10 ng ml^−1^) in response to *β*43–63. (**C**) HuDMEC migration across a collagen-coated filter in response to medium alone (control) or medium containing 10 ng ml^−1^ VEGF. (**D**) HuDMEC proliferation in response to medium alone (control or medium containing VEGF (10 ng ml^−1^)). ^*^P<0.05 with respect to relevant control group. All data are the average of three repeat experiments.

Our previous experiments have shown that 1 *μ*M FgnE (the parent molecule of *β*43–63) is cytotoxic for GF-activated ECs (Bootle-Wilbraham *et al*, 2000). We therefore wished to ascertain whether the inhibitory effects of *β*43–63 on tubule formation was due to a similar cytotoxic effect in culture. Figure 3A shows that *β*43–63 slightly abrogated the pro-survival effect of VEGF on ECs, inducing a marginal (<10%) increase in late-stage apoptosis/necrosis in VEGF-activated ECs. This effect was not seen when scrambled *β*43–63 was used. To investigate whether this cytotoxic effect was mediated through the PI3K/Akt pathway, we undertook western blotting analysis of phosphorylated Akt. Although VEGF stimulated Akt phosphorylation, this was only marginally (nonsignificantly) inhibited by *β*43–63 (Figure 3B) and unaffected by *β*SC.

These findings suggested that the marked inhibitory effects of *β*43–63 in the tubule formation assay (Figure 2) were not due to induction of extensive EC death. Moreover unlike FgnE and alphastatin, *β*43–63 had no effect on either EC migration or proliferation (Figure 3C and D), suggesting an alternative mechanism of action for *β*43–63.

**Figure 4:**
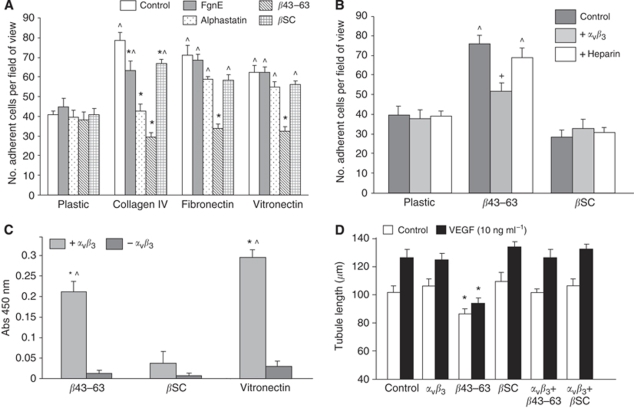
*β*43–63 markedly inhibits adhesion of activated human ECs to various extracellular matrix proteins *in vitro*: possibly by binding to *α*_v_*β*_3_. (**A**) Effects of *β*43–63 and related fibrinogen fragments on HuDMEC adhesion to three different ECM proteins. ^*P*<0.05 with respect to relevant plastic group. ^*^P<0.05 with respect to relevant control group. (**B**) Effects of exogenous soluble *α*_v_*β*_3_ or heparin on adhesion of HuDMECs to plates coated with *β*43–63 or *β*SC. ^*P*<0.05 with respect to relative plastic group. ^+^*P*<0.05 with respect to relevant control (untreated) group. (**C**) Direct binding of *α*_v_*β*_3_ to *β*43–63, *β*SC (negative control) or vitronectin (positive control) in a solid-phase assay. ^*^P<0.01 with respect to *β*SC negative control. ^*P*<0.003 with respect to-*α*_v_*β*_3_ control. (**D**) Effects of pre-incubating *β*43–63 or scrambled *β*43–63 with recombinant *α*_v_*β*_3_ before addition to the Matrigel assay. ^*^P<0.05 with respect to relative control group. All data are means±s.e.m. Data are representative of three replicate experiments.

We then investigated whether *β*43–63 might inhibit angiogenesis by inhibiting VEGF-activated adhesion of ECs to ECM proteins (an important step in the angiogenic pathway (Eliceiri, 2001)), namely collagen IV, fibronectin and vitronectin (Hynes, 2007; Mundel and Kalluri, 2007). Endothelial cells were pre-incubated with test peptides and VEGF for 1 h, and then allowed to adhere for a further hour (Delaney *et al*, 2006). Whereas both FgnE and alphastatin significantly (*P*<0.05) inhibited EC adhesion to collagen IV, *β*43–63 significantly (*P*<0.05) inhibited EC adhesion to all three ECM proteins. This effect was not seen with scrambled *β*43–63 (Figure 4A).

Endothelial cells adhere to the ECM largely through cell-surface heparin sulphate proteoglycans and integrins and *α*_v_*β*_3_ is the only integrin to bind to all three of ECM proteins used here. We therefore investigated whether *β*43–63 might bind to heparin or *α*_v_*β*_3_ and thus block EC adhesion to the ECM. Activated EC were seen to adhere to *β*43–63 in a manner that was significantly (*P*<0.05) but not completely blocked by the addition of exogenous recombinant human *α*_v_*β*_3_, a noncovalent heterodimer containing the ligand binding extracellular portion of human *α*_v_*β*_3_ (Figure 4B). Moreover direct binding studies confirmed that recombinant *α*_v_*β*_3_ binds to vitronectin (a positive control) and *β*43–63 in a solid-phase assay (Figure 4C). In contrast heparin did not significantly affect EC adhesion to *β*43–63 (Figure 4B). To further investigate the possibility of *β*43–63 mediating its effects by *α*_v_*β*_3_, we incubated *β*43–63 with recombinant *α*_v_*β*_3_ before use in the Matrigel assay. In this experiment recombinant *α*_v_*β*_3_ alone had no effect on tubule formation, but completely ablated the *β*43–63 inhibition of tubule formation (Figure 4D).

**Figure 5:**
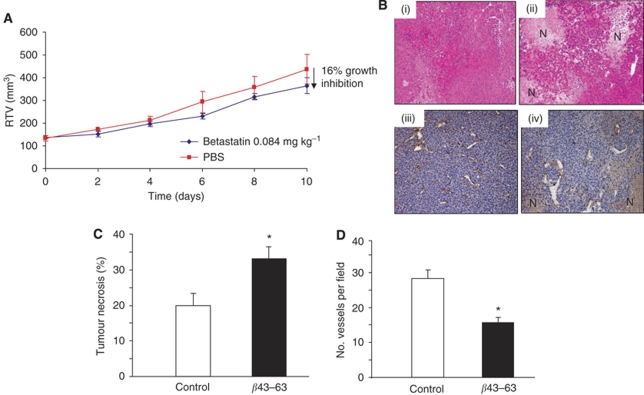
Effects of *β*43–63 on CaNT mammary tumours *in vivo*: *In vivo* effects of *β*43–63 on (**A**) the volume and (**B**) histological appearance of CaNT tumours grown in mice. Data are shown as mean±s.e.m. (**B**) Tumours were excised from control (i, iii) or *β*43–63 treated (ii, iv) mice and general morphology/histology was examined at low magnification (i, ii) or stained with an anti-murine CD31 antibody and viewed at higher magnification (iii, iv). Cells in control tumours exhibited a compact regular morphology (i) with many small patent vessels in the viable regions lined with a continuous single layer of endothelial cells (iii). By contrast, *β*43–63 treated tumours exhibited an irregular overall morphology with increased levels of necrosis (N; ii, iv) and relatively few large vessels in the viable regions (iv). Graphs showing (**C**) percentage tumour necrosis and (**D**) CD31 vessel counts per field of view in tumours. All data are means±s.e.m. ^*^P<0.05 with respect to control tumours.

Finally, we investigated whether *β*43–63 could inhibit tumour growth and angiogenesis *in vivo* using the CaNT murine mammary adenocarcinoma model. Control tumours grew steadily over the 10-day injection period, whereas *β*43–63 treated tumours showed nonsignificantly slower growth (being 16% smaller by day 10; Figure 5A). The experiment could not be extended beyond this point as the control tumours started to ulcerate and the mice had to be killed. *β*43–63 injections were well tolerated *in vivo* with no significant effect on body weight or the general well being of the animals. A significant reduction in the number of vessels (*P*<0.02) within the *β*43–63 treated tumours was observed (Figure 5B and D). Moreover, a central area of necrosis was evident in both control and *β*43–63 treated tumours, but was significantly (*P*<0.03) larger in *β*43–63 treated mice (Figure 5B and C).

## Discussion

Complex interactions between microvascular ECs and factors in the local microenvironment (eg GFs, ECM constituents) regulate angiogenesis. In response to such signals, ECs degrade surrounding matrices, proliferate, migrate and fully differentiate to form new capillary tubules. *In vitro* models of these various steps are invaluable in gaining insights into the mode of action of newly identified angiogenesis regulators. We have used an array of such assays to fully characterise the anti-angiogenic effects of a peptide derived from human Fgn, *β*43–63. We show that although *β*43–63 inhibited EC differentiation as measured by tubule formation in response to multiple GFs, it had no effect on their migration or proliferation, and only a marginal effect on viability. It also inhibited the adhesion of VEGF-activated ECs to a number of angiogenesis-regulating ECM proteins – an effect involving blockade by the *β*43–63 peptide of *α*_v_*β*_3_ on the surface of activated ECs.

Interestingly, although *β*43–63 inhibited the effects of multiple GFs (VEGF, PDGF, HGF and FGF2) on ECs, it had much less of an effect on their activation by EGF. The overlapping signalling pathways used by VEGF, PDGF, HGF and FGF2 suggests that *β*43–63 may inhibit ECs by the suppression of signalling molecules that are common to these four GFs, such as PI3K/Akt and/or FAK/Paxillin (focal adhesion). Alteration of FAK/Paxillin association and phosphorylation by *β*43–63 would be highly likely to result in reduced GF-induced migration (Saito *et al*, 2008) – an effect that was not seen with the peptide (Figure 3B). The marginal reduction in cell survival seen with *β*43–63 (Figure 3A), and the minimal effect seen on Akt phosphorylation suggests that it also does not profoundly modulate the PI3K/Akt pathway in ECs (which is known to regulate EC survival and apoptosis (Kucharzewska *et al*, 2009)). Further studies are now warranted to identify the point of interaction between the signalling pathway activated by *β*43–63 receptor binding and those activated by VEGF, PDGF, HGF and FGF2.

The fact that *β*43–63 inhibited adhesion of VEGF-activated ECs to all three ECM proteins used suggests that this may represent at least part of the mechanism by which it inhibits tubule formation by VEGF-activated ECs (as GF-reduced Matrigel contains 30% collagen IV; http://www.bdbiosciences.com). However, blocking of adhesion is unlikely to be the only mechanism involved as *β*43–63 failed to inhibit migration of ECs across collagen type I or IV coated filters in our migration assays, and had a pro-apoptotic effect on VEGF-stimulated ECs in the absence of any ECM proteins.

Endothelial cells express a wide array of integrins that mediate their interaction with, and activation by, ECM components and GFs. Integrin *α*_v_*β*_3_ is the only known integrin to bind to all three ECM proteins tested in our adhesion assays (Mizejewski, 1999; Rüegg and Mariotti, 2003), and moreover *α*_v_*β*_3_ is known to interact with the receptors for all the GFs tested and increases angiogenesis when bound to ECM protein ligands (Soldi *et al*, 1999; Borges *et al*, 2000; Sahni and Francis, 2004; Kemp *et al*, 2006; Ellis *et al*, 2007). However, when soluble ligands bind/activate *α*_v_*β*_3_, the ability of various GFs (eg VEGF, FGF2, PDGF and HGF) to activate their own receptors is inhibited; for example, interaction of soluble ligands with *α*_v_*β*_3_ can reduce VEGF-stimulated VEGF-R2 tyrosine phosphorylation (Soldi *et al*, 1999). Interestingly, although EGFR does bind to *α*_v_*β*_3_, activation of HuDMECs by EGF does not require the binding of *α*_v_*β*_3_ to EGFR (Ellis *et al*, 2007), which may explain why *β*43–63's effects on EGF are more limited compared to the other GFs (ie where *α*_v_*β*_3_ binding to receptors is required for full activation; Borges *et al*, 2000). We therefore hypothesised that interaction of *β*43–63 with *α*_v_*β*_3_ on the surface of ECs could explain its ability to inhibit multiple GF pathways stimulating EC differentiation. Moreover, some studies suggest that when *α*_v_*β*_3_ is occupied by a soluble ligand (such as *β*43–63) it can mediate pro-apoptotic signals (Maeshima *et al*, 2001), which may explain the slight increase in apoptosis with *β*43–63.

The *α*_v_*β*_3_ integrin is known to bind to the RGD^572−574^ sequence on the *α* chain of whole Fgn (Yokoyama *et al*, 1999). However, *β*43–63 is derived from the *β* rather than the *α* chain of Fgn, and does not contain an RGD or RGD-like sequence (Figure 1). It is now recognised that proteins and peptides can bind to *α*_v_*β*_3_ by non-RGD regions; for example, tumstatin, an inhibitor of tumour angiogenesis induces apoptosis of ECs by an *α*_v_*β*_3_ integrin-dependent manner (Maeshima *et al*, 2002), using non-RGD sequences (Maeshima *et al*, 2000), although *β*43–63 bears no homology with the tumstatin *α*_v_*β*_3_ binding regions. Our experiments show recombinant *α*_v_*β*_3_ binds directly to *β*43–63, partially inhibits the binding of HuDMEC to *β*43–63, and incubation of recombinant *α*_v_*β*_3_ with *β*43–63 before the Matrigel experiment ablates the ability of *β*43–63 to inhibit EC differentiation/tubule formation. Therefore, taken together, our data suggest that one possible receptor for *β*43–63 could be a non-RGD binding sequence on the *α*_v_*β*_3_ integrin.

Although studies investigating Fgn have shown that *β*43–57 (contained within *β*43–63) is a heparin binding site (Yakovlev *et al*, 2003), and heparin sulphate proteoglycans are used as co-receptors for the GFs (except EGF) (Neufeld *et al*, 1994; Chua *et al*, 2004; Kemp *et al*, 2006), binding studies have shown that the affinity of *β*43–57 for heparin is relatively low compared to dimerised *β*15–66 peptides (Yakovlev *et al*, 2003), suggesting that dimerisation is essential for high-affinity HS binding. As the *β*43–63 peptide used in our study is not dimerised, it is unlikely that such binding would mediate the effects seen with *β*43–63. Indeed our data show that heparin had no effect on the binding of HuDMECs to *β*43–63 (Figure 4B) suggesting that heparin binding is not involved in mediating *β*43–63 activity.

Interestingly, in contrast to FgnE and alphastatin that inhibit tumour growth by causing vascular damage and an increase in intravessel thrombosis within the tumours rather than by anti-angiogenic mechanisms (Brown *et al*, 2002; Staton *et al*, 2004), *β*43–63 caused a significant decrease in tumour vascularisation (ie was anti-angiogenic) without any evidence of thrombosis. This decrease in vessel counts could account for the increase in tumour necrosis, and decrease in tumour growth and suggests that should the control tumours not have ulcerated and therefore the experiment be continued beyond 10 days treatment, a greater effect on tumour growth would have been observed. The effects observed on blood vessels is similar to that reported using known *α*_v_*β*_3_ antagonists (RGD-containing drugs) where vessel counts were reduced, but there were no observed effects on vascular thrombosis (Park *et al*, 2008), adding further evidence that *α*_v_*β*_3_ may be a receptor for *β*43–63.

In sum, our data show for the first time that a novel, 20-amino-acid peptide derived from the *β* chain of human Fgn, has a unique ability to inhibit the angiogenic responses of ECs to multiple GFs such as VEGF, FGF2, PDGF and HGF *in vitro*, and to inhibit tumour vascularisation *in vivo*. This appears to involve, in part, the binding of *β*43–63 to *α*_v_*β*_3_ integrin and the reduced ability of activated ECs to adhere to various ECM proteins. Further studies are now warranted to better understand the mode of action of this new agent so that its efficacy in anti-angiogenic therapies can be maximised.
